# First report of molecular taxonomic analyses of European beaver metazoan parasites from Hungary

**DOI:** 10.1007/s00436-022-07547-y

**Published:** 2022-05-24

**Authors:** Sándor Szekeres, Dávid Czabán, Nóra Takács, Zoltán Széll, András Gubányi, Jenő Kontschán, Sándor Hornok, Tamás Sréter

**Affiliations:** 1 Department of Parasitology and Zoology, University of Veterinary Medicine, Budapest, Hungary; 2 Department of Zoology, Hungarian National History Museum, Budapest, Hungary; 3 National Reference Laboratory for Parasitology, Fish and Bee Diseases, National Food Chain Safety Office, Budapest, Hungary; 4 Plant Protection Institute, Centre for Agricultural Research, ELKH, Budapest, Hungary; 5 Hungarian Natural History Museum, Main Office, Budapest , Hungary

**Keywords:** Eurasian beaver, Reintroduction, Parasites, *Stichorchis*, *Schizocarpus*, *Platypsyllus*

## Abstract

European beaver (*Castor fiber* L. 1758) is the biggest rodent species living in Europe. Beavers are semi-aquatic animals; they are defecating directly into the water; thus, they have an important role in spreading parasites related to water (e.g., protozoa and flukes). The first specimens of this once extinct rodent species in Hungary turned up in Szigetköz (upper flow of the Hungarian Danube) in 1991 dispersed from Austria. The reintroduction to Hungary started in 1996, and the population slowly increased in number up to around 4000 individuals, but the knowledge about their parasites is lacking. This is the first report on the metazoan parasites of beavers in Hungary and their molecular taxonomy. In the 5-year study, 47 beavers were trapped in four locations and euthanized with permission. Three different metazoan parasites were collected: larvae and adults of *Platypsyllus castoris* beetles, nymphs and adults of *Schizocarpus* sp. mites and eggs and adults of *Stichorchis subtriquetrus* flukes. From these three parasite species, molecular taxonomic studies were also carried out. The low number of metazoan parasites species detected in Hungarian beavers compared to other European countries (e.g., Poland) might be attributed to host population bottleneck effect during reintroduction. As parasites represent a significant component of the biodiversity and ecosystem, the conservation efforts should focus not only on host species but also on their parasites.

**Supplementary Information:**

The online version contains supplementary material available at 10.1007/s00436-022-07547-y.

## Introduction 

Eurasian or European beaver (*Castor fiber*) is the largest rodent species in Europe, building dams and burrows in aquatic habitats of Eurasia (Halley et al. 2021). Beavers are obligate herbivores consuming tree bark, aquatic plants, grasses, and sedges. According to their semi-aquatic lifestyle, they are defecating directly into the water; thus, they have an important role in spreading parasites related to water, or considered water-borne (e.g., protozoa and flukes).

Beavers are protected mammals in Hungary with the growing population. The first specimens of this once extinct animal turned up in Szigetköz (upper flow of the Hungarian Danube) in 1991 dispersed from Austria (Czabán and Gruber 2018). The reintroduction to Hungary started in 1996 from Bavaria (Bajomi 2011) by World Wildlife Found (WWF) Hungary, and the population slowly increased in number up to around 4000 individuals for 2016 (Czabán and Gruber 2018).

Protozoon parasites present in European beavers are *Cryptosporidium*, *Giardia* (Bystrianska et al. 2021), and *Eimeria* (Goodman et al. 2012) spp. Among endoparasite helminth species, the flatworms *Psilotrema castoris*, *Stichorchis subtriquetrus* (Demiaszkiewicz et al. 2014), *Echinococcus multilocularis*, *Taenia martis* (Campbell-Palmer et al. 2015), *Fasciola hepatica* (Shimalov and Shimalov VT 2000); the nematode *Travassosius rufus* (Bystrianska et al. 2021), *Trichostrongylus capricola* (Demiaszkiewicz et al. 2014), *Calodium hepaticum* (Mészáros and Kemenes 1973; Fuehrer 2014), *Trichinella spiralis* (Różycki et al. 2020) and *Trichinella britovi* (Seglina et al. 2015) were found in *C. fiber*. Among ectoparasites, there are fur mites from the genus *Schizocarpus*, *Demodex* follicule mites (Izdebska et al. 2016); two hard tick species *Ixodes hexagonus* (Haitlinger 1991) and *Ixodes apronophorus* (Kadulski 1998) and a unique epidermal tissue feeding beetle, *Platypsyllus castoris* (Åhlen et al. 2021). Beavers can serve as a host for a wide range of rodent-related pathogens as well. Despite the fact the number of beavers in Hungary and reports and articles dealing with the beavers’ eco-engineering service are increasing, the information regarding the parasitological status of these rodents is limited.

The aim of this study was to determine the helminth and arthropod parasites species of reintroduced wild beavers in Hungary.

## Materials and methods

### Classic parasitological methods

**Fig. 1:**
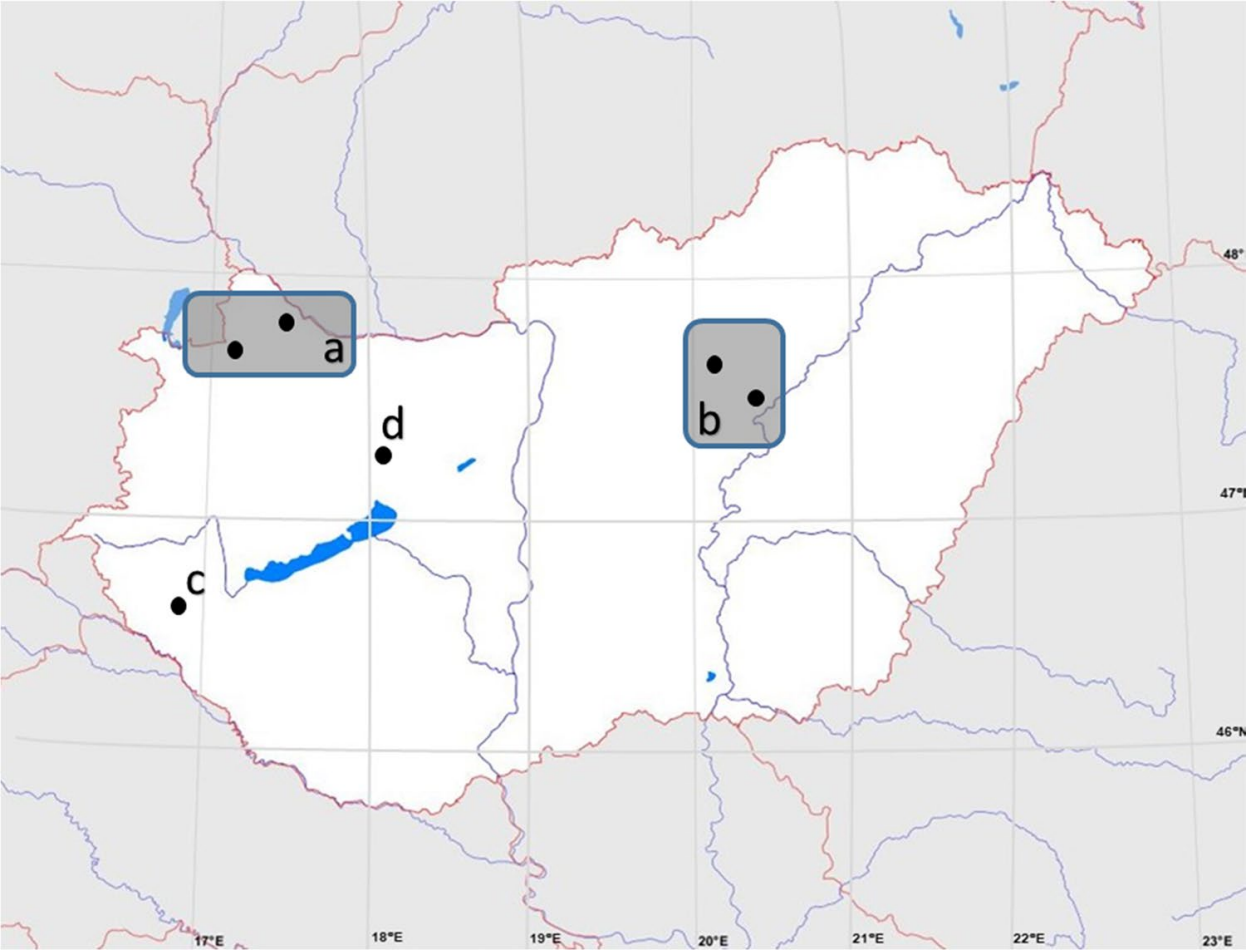
*Castor fiber* trapping sites in Hungary 2017–2021. **a** Győr-Moson-Sopron county 13 individuals; **b** Jász-Nagykun-Szolnok county 26 individuals; **c** Zala county 6 individuals; location; **d** Veszprém county 2 individuals

Forty-seven beavers were caught with permission from the authorities (see permission numbers in the Ethical consent section) from 2017–2021. Individuals used in this research were in different conditions (full body, skinned headless torso, only hide or just gastrointestinal tract). Because of value and uniqueness of the carcasses multiple research projects used some part of these animals. The beavers were collected in multiple locations (Győr-Moson-Sopron, Jász-Nagykun-Szolnok, Zala and Veszprém counties) (Fig. 1) and stored without fixation on 4 °C or frozen until the autopsy, and the collection of the parasites was carried out in the National Reference Laboratory for Parasites, Fish and Bee Diseases of the National Food Chain Safety Office and in the Department of Parasitology and Zoology, University of Veterinary Medicine. The whole-body surface of the beavers was examined carefully for the presence of ectoparasites. Flea comb was also used to examine the fur. Lungs and livers of the beavers were dissected and examined for helminths macroscopically and using stereomicroscope. The stomach, small intestine, caecum, and colon of all beavers were separated and cut longitudinally. The gastrointestinal mucosa and content were collected and tested by sedimentation and counting technique. The colon content was tested by flotation technique for the presence of nematode eggs and coccidium oocysts and by sedimentation technique for the presence of trematode eggs. Muscle samples were collected from the lower forelimb, diaphragm, and tongue. More than 10 g of muscle tissue trimmed of fat and fascia were collected from each animal. All samples were digested individually according to the magnetic stirrer method for pooled sample digestion. Collected parasites were stored in 70% ethanol until morphological examination under stereomicroscope (Nikon SMZ-2 T, Japan). *Platypsyllus castoris* and *S. subtriquetrus* were identified to the species level based on descriptions (Peck 2006; Máca et al. 2015). However, *Schizocarpus* mites could be identified only on genus level, because after clearance in lactic acid, morphological characters did not correspond to any of the species in standard keys (Fain and Lukoschus 1985; Bochkov and Saveljev 2012a, b).

### DNA extraction

DNA were extracted from random parasites (individually: helminths and beetles, pooled sample from 20 mite individuals) with ISOLATE II Genomic DNA Kit (Meridian Bioscience Inc., Cincinnati, USA) and QIAamp DNA Mini Kit (Qiagen GmbH, Hilden, Germany). The extraction was performed following the manufacturers protocol after evaporating the ethanol from the samples and a three-step washing method (water with detergent and 2X bidistilled water).

### Molecular methods

Conventional PCR reactions were used with the primer pairs: STH18SF and STH18SR to amplify a ~ 1800 bp long fragment from the 18S rRNA gene of *S. subtriquetrus* (Campbell-Palmer et al. 2009); LCO1490 and HCO2198 to amplify a ~ 710 bp long fragment from the cytochrome *c* oxidase subunit I (COI) gene of *P. castoris* (Folmer et al. 1994); and bcdf05 and bcdR04 to amplify a ~ 710 bp long fragment from the cytochrome *c* oxidase subunit I (COI) gene *Schizocarpus* mites (Dabert et al. 2010). The PCR reactions were modified with the following conditions, 5 µl of extracted DNA were added to 20 µl of reaction mixture containing 1 U of HotStar Taq Plus DNA Polymerase (5U/µl) (QIAGEN, Hilden, Germany), 0.5 µl of dNTP Mix (10 mM), 0.5 µl of each primer (50 µM), 2.5 µl of 10 × Coral Load PCR buffer (15 mM MgCl_2_ included), and 15.8 µl of distilled water. In the *Schizocarpus* cytochrome *c* oxidase subunit I (COI) reaction, we used 1 µl of extra MgCl_2_ and 14.8 µl of distilled water to the reaction mixture. The primer sequences and the thermocycling profile are presented in Table 1.

**Table 1:** Primers and cycle conditions of conventional PCRs used in this study

Species	Primer name	Primer sequence	Thermal profile	Reference
*Stichorchis subtriquetrus*	STH18F STH18R	5′-CTA AGT ACA TAC CTT TAA ACG G-3′ 5′-CTC TAA ATG ATC AAG TTT GG-3′	95 °C for 5 min; 40x (94 °C for 30 s, 55 °C for 30 s, 72 °C for 1 min); 72 °C for 7 min	Campbell-Palmer et al. 2009
*Platypsyllus castoris*	LCO1490 HCO2198	5′-GGT CAA CAA ATC ATA AAG ATA TTG G-3′ 5′-TAA ACT TCA GGG TGA CCA AAA AAT CA-3′	95 °C for 5 min; 40x (94 °C for 40 s, 48 °C for 1 min, 72 °C for 1 min); 72 °C for 10 min	Folmer et al. 1994
*Schizocarpus* mites	bcdf05 bcdR04	5′-TTT TCT ACH AAY CAT AAA GAT ATT GC-3′ 5′-TAT AAA CYT CDG GAT GNC CAA AAA A-3′	95 °C for 5 min; 40x (94 °C for 45 s, 50 °C for 1 min, 72 °C for 1 min); 72 °C for 10 min	Dabert et al. 2010

### Gel electrophoresis and sequencing

All PCR products were electrophoresed in 1.5% agarose gel (100 V, 50 min), stained with ethidium bromide and visualized under ultra-violet light.

Positive PCR products of *S. subtriquetrus* and *P. castoris* were cleaned with Wizard® SV Gel and PCR Clean-Up System, Promega (Madison, USA) and sequenced by LGC Genomics GmbH (Berlin, Germany). Positive PCR product of *Schizocarpus* sp. were cleaned and sequenced by BIOMI Ltd. (Gödöllő, Hungary).

### Phylogenetical analysis

Sequences were manually edited with BioEdit (Hall 1999), aligned, and compared to reference GenBank sequences by nucleotide BLASTn program (https://blast.ncbi.nlm.nih.gov). All sequences retrieved from GenBank and included in the phylogenetic analysis had 97–100% coverage (i.e., aligned with a near-identical length and starting position) as sequences from this study. This dataset was resampled 1000 times to generate bootstrap values. Phylogenetic analysis was conducted by using the maximum likelihood method and GTR (mite) and Kimura (fluke) model according to the best-fit selection with the program MEGA 7.0 (Kumar et al. 2016).

## Results and discussion

Altogether, 47 beavers were collected from 2017 to 2021. In this study, we used all the digestive tracts and 27 hide of the carcasses (all the available). In 38 (80.85%, CI: 66.74–90.85%) gastrointestinal tracts, 1840 *S. subtriquetrus* flukes were presented (maximum intensity 400, mean intensity 48 individual/beaver), and we also found fluke eggs in the feces in additional 15 cases (examined by sedimentation method). DNA extracted from the adult flukes (GenBank accession number: OK040064) showed highest (99.4%) similarity with a *S. subtriquetrus* sequence (AY245769.1) and high similarity (99.3–98.3%) with nonspecified Paramphistomidae sequences (AY222110.1; FJ550131.1). The beaver fluke is the most frequent parasite reported in beavers in wide geographic range from North America to Eurasia (Máca et al. 2015; Bystrianska et al. 2021). These parasites are usually found in the caecum of the host. Beaver flukes were the most prevalent and the most numerous parasites in our study (80.85%, 1840 individuals).

**Fig. 2:**
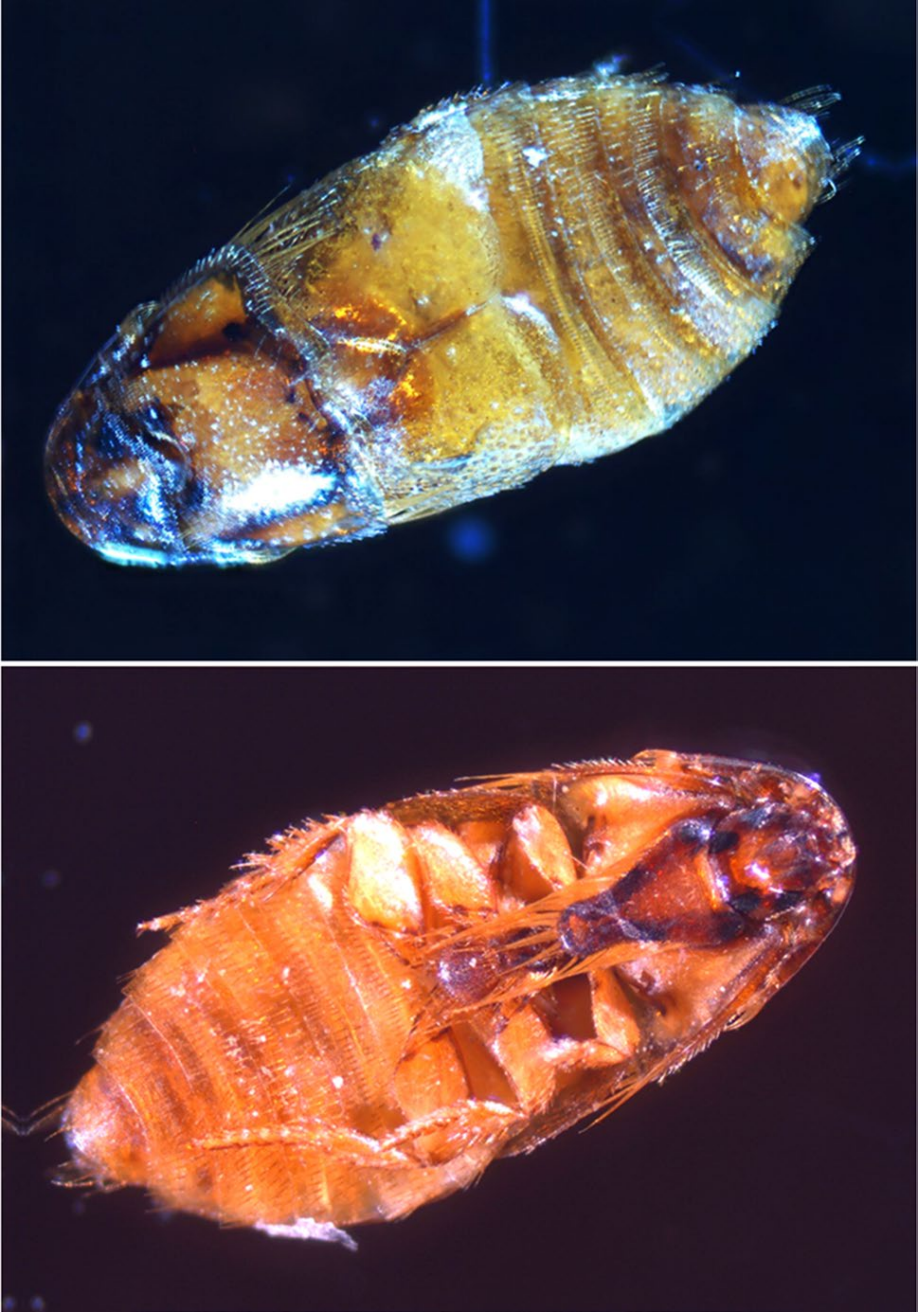
Dorsal and abdominal view of *Platypsyllus castoris* adult removed from *Castor fiber* in Hungary

Regarding the ectoparasite load, two arthropod species, *P. castoris* beetles and *Schizocarpus* mites, were also detected. We have found in total 167 beaver beetle (Fig. 2) on 13 (27.66%, CI: 15.62–42.64%) beavers with the maximum intensity 46 on one host. In four cases (8.51%, CI: 2.37–20.38%), we also found five *P. castoris* larvae on the carcasses. *Platypsyllus castoris* DNA extracted from these specimens (accession number: OK039272) have 100% similarity with a *P. castoris* sequence (accession number: KM448659.1) from Germany (Hendrich et al. 2015).

**Fig. 3:**
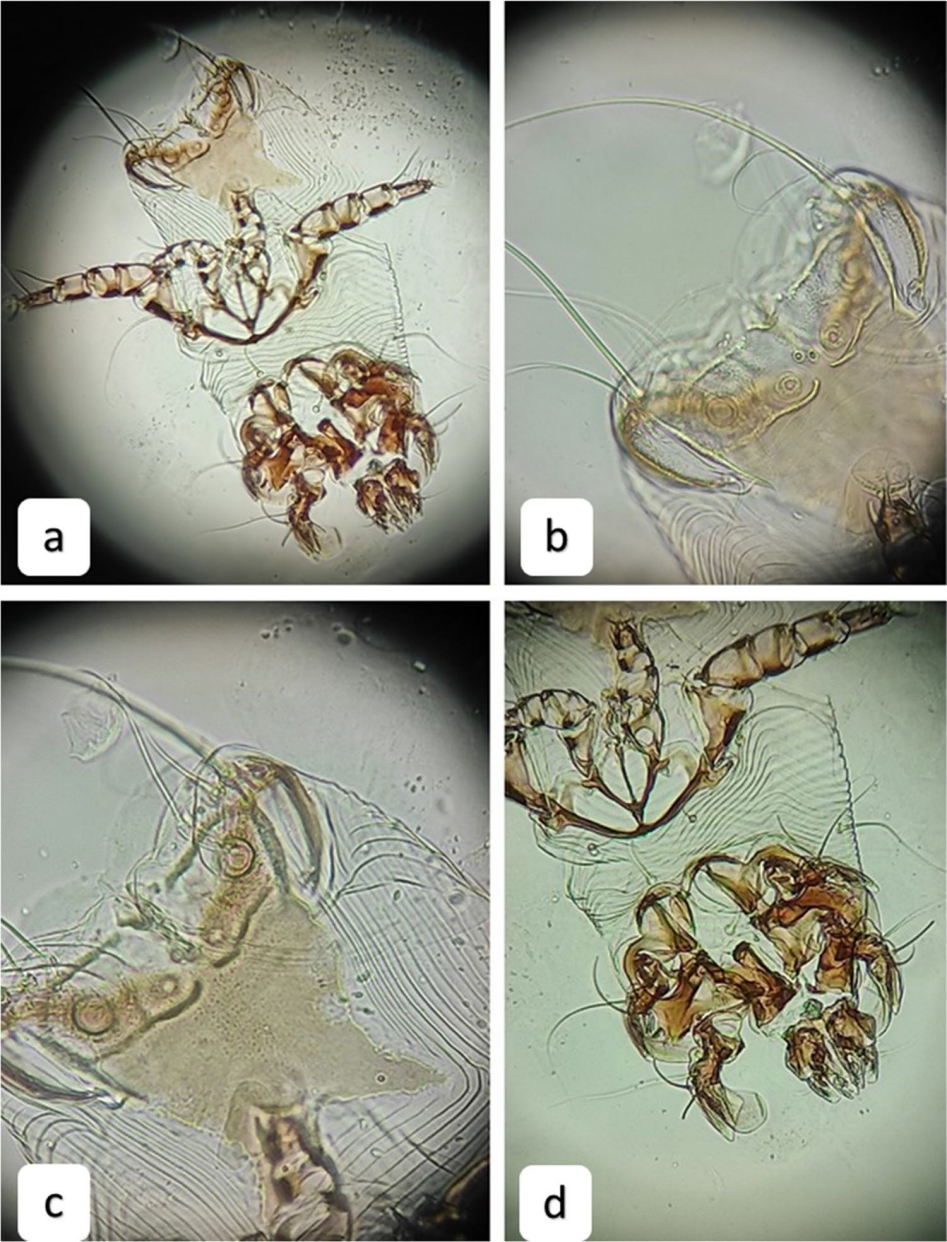
Characteristics of *Schizocarpus* sp. adult male mite from *Castor fiber* in Hungary, **a** habitus in ventral view, **b** ventral opisthosoma with the suckers and the setae, **c** opisthosoma and the opisthonotal shield, **d** anterioventral part of the mite with the mouthparts and legs in higher magnification

*Schizocarpus* mites (Fig. 3) were found on 5 beavers in the ears. However, *Schizocarpus* mites could only identified on genus level, because after clearance in lactic acid, morphological characters did not correspond to any of the species in standard keys (Fain and Lukoschus 1985; Bochkov and Saveljev 2012a, b). DNA extracted from these mites (OK047144) show around 88% similarity with other astigmated mites from the parvorder Psoroptidia. The new *Schizocarpus* sp. sequence has 88.49% similarity with another *Schizocarpus* sp. sequence from 2010 (accession number: GQ864344.1) (Dabert et al. 2010). Reports of *Schizocarpus* sp. mites on beavers are scarce, and the reports mainly focus on describing a new species based on few individuals. In our study, more than 200 individuals were collected from the ears of the beavers (Supplementary video).

*Stichorchis subtriquetrus* flukes and *Schizocarpus* sp. mites are host-specific only occurring on Eurasian and American beavers (*C. fiber* and *C. canadensis*) (Bystrianska et al. 2021). *Platypsyllus castoris*, the beaver beetle, is also beaver-related, but it was also found on otters (Belfiore 2006; Pushkin 2010). These parasites are new species in Hungary; thus, the successful reintroduction of beavers also added at least three surplus parasites species to the Hungarian fauna.

To report the *P. castoris* load of beavers accurate is challenging because these motile insects can leave the host quickly in huge numbers during the handling of the host, usually in the time where it is in the trap or not long after the trapping. Thus, our data (27.66%, 167 individuals) are an underestimation of a normal Hungarian beaver’s beetle load, which was almost 100% as reported by our colleague responsible for beaver collection and transport.

**Fig. 4:**
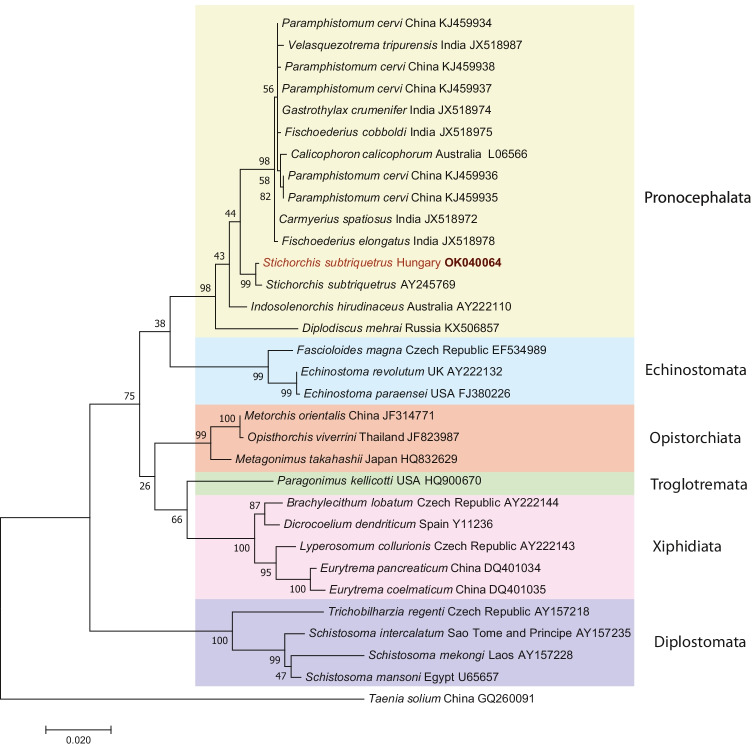
Phylogenetic tree of *Stichorchis subtriquetrus* beaver fluke and related flukes based on the 18S rRNA gene. The tree was generated with the maximum likelihood method and Kimura model in MEGA 7.0. Nucleotide sequences obtained in this study are indicated in red. Branch lengths represent the number of substitutions per site inferred according to the scale shown

**Fig. 5:**
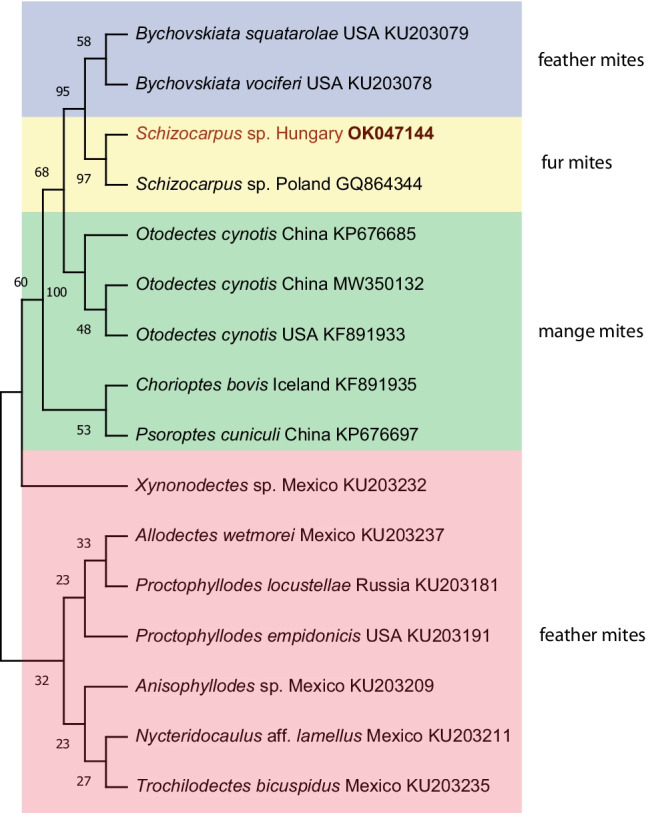
Phylogenetic tree of *Schizocarpus* beaver fur mites and related astigmated mites based on the cytochrome *c* oxidase subunit I (COI) gene. The tree was generated with the maximum likelihood method and GTR model in MEGA 7.0. Nucleotide sequences obtained in this study are indicated in red. Branch lengths represent the number of substitutions per site inferred according to the scale shown

We used the fluke and the mite sequences to generate an informative phylogenetical tree of parasitic species. The Hungarian *S. subtriquetrus* sequence was paired with beaver fluke sequence (AY245769) from North America (non-specified location). This Stichochiinae branch is a sister branch of a larger group of flukes mixed from the Paramphistominae and the Gasterochilinae subfamilies in the Pronocephalata group (Fig. 4). The mite tree is more interesting because the *Schizocarpus* fur mites, and mange mites are separating the feather mites in two non-monophyletic groups (Fig. 5). Based on Fig. 5, the *Schizocarpus* fur mites are closer related to *Bychovskiata* feather mites than the also mammalian-related mange mite group.

This research is one of the few articles dealing with the field of beaver parasites, but the first which presents the sequences of all collected parasite species and their phylogenetical relations of two species.

Compared to endemic European beaver population (Romashov 1969), the number of the taxa found in Hungary is low. In Hungary, only one beaver-specific worm species was found, unlike in Poland, where three of them were reported (Demiaszkiewicz et al. 2014). In the Hungarian beavers, only a single endoparasite taxon was detected. The low number of parasite species found in Hungarian beavers might be attributed to the host population bottleneck during the reintroduction. The Hungarian population was almost exclusively originated from Bavaria (Bajomi 2011). This bottleneck effect might have resulted the loss of more than one of the beaver parasites as it was proposed by Åhlen et al. (2021) in Sweden. As parasites represent significant component of the biodiversity and ecosystem, the conservation efforts should focus not only on host species but also on their parasites. The non-human pathogenic parasites can be equally important for the fauna (Jørgensen 2015); nevertheless, the reintroduction of highly pathogenic zoonotic parasites should be avoided. As beavers can serve as intermediate hosts of *Echinococcus multilocularis* and Hungarian beavers originated from an endemic region (Bavaria), it cannot be excluded that these rodents played a role in spreading of *E. multilocularis* to Hungary (Sréter et al., 2004). Interestingly, the parasite first distributed along the watershed area of the River Danube in northern Hungary.

## Supplementary Information

Below is the link to the electronic supplementary material.

Supplementary file1 (MP4 8297 KB)

## Data Availability

The sequences generated in the study have been deposited in the GenBank database under the accession number OK040064 for the 18S rRNA gene of *S. subtriquetrus*, respectively, and OK039272 and OK047144 for the COI gene of *P. castoris* and *Schizocarpus* sp.
